# Development of a Novel Restrictive Medium for *Monascus* Enrichment From Hongqu Based on the Synergistic Stress of Lactic Acid and Ethanol

**DOI:** 10.3389/fmicb.2021.702951

**Published:** 2021-06-21

**Authors:** Kangxi Zhou, Li Wu, Guimei Chen, Zhibin Liu, Xinze Zhao, Chen Zhang, Xucong Lv, Wen Zhang, Pingfan Rao, Li Ni

**Affiliations:** ^1^College of Chemical Engineering, Fuzhou University, Fuzhou, China; ^2^Fujian Center of Excellence for Food Biotechnology, Institute of Food Science and Technology, Fuzhou University, Fuzhou, China

**Keywords:** *Monascus* enrichment, restrictive medium, Hongqu, lactic acid, ethanol

## Abstract

Hongqu is a famous fermented food produced by *Monascus* and has been used as food coloring, wine starters and food additives for thousands of years in China. Excellent *Monascus* strain is an important prerequisite for producing high-quality Hongqu. However, the isolation of *Monascus* pure culture from Hongqu samples is time-consuming and laborious because it is easily interfered by other microorganisms (especially filamentous fungi). Therefore, the development of restrictive medium for *Monascus* enrichment from Hongqu is of great significance for the preparation and screening of excellent *Monascus* strains. Results of this study showed that *Monascus* has good tolerance to lactic acid and ethanol. Under the conditions of tolerance limits [7.5% lactic acid (v/v) and 12.0% ethanol (v/v)], *Monascus* could not grow but it still retained the vitality of spore germination, and the spore activity gradually decreased with the increasing concentrations of lactic acid and ethanol. More interestingly, the addition of lactic acid and ethanol significantly changed the microbial community structure in rice milk inoculated with Hongqu. After response surface optimization, *Monascus* could be successfully enriched without the interference of other microorganisms when 3.98% (v/v) lactic acid and 6.24% (v/v) ethanol were added to rice milk simultaneously. The optimal enrichment duration of *Monascus* by the restrictive medium based on the synergistic stress of lactic acid and ethanol is 8∼24 h. The synergistic stress of lactic acid and ethanol had no obvious effects on the accumulation of major metabolites in the progeny of *Monascus*, and was suitable for the enrichment of *Monascus* from different types of Hongqu. Finally, the possible mechanisms on the tolerance of *Monascus* to the synergistic stress of lactic acid and ethanol were preliminarily studied. Under the synergistic stress of lactic acid and ethanol, the cell membrane of *Monascus* defends against lactic acid and ethanol into cells to some extent, and the superoxide dismutase (SOD), catalase (CAT) and glutathione peroxidase (GSH-Px) activities of *Monascus* were higher than those of other fungi, which significantly reduced the degree of lipid peroxidation of cell membrane, while secreting more amylase to make reducing sugars to provide the cells with enough energy to resist environmental stress. This work has great application value for the construction of *Monascus* strain library and the better development of its germplasm resources.

## Graphical Abstract

The restrictive medium containing lactic acid and ethanol enabled the enrichment of *Monascus* sp.

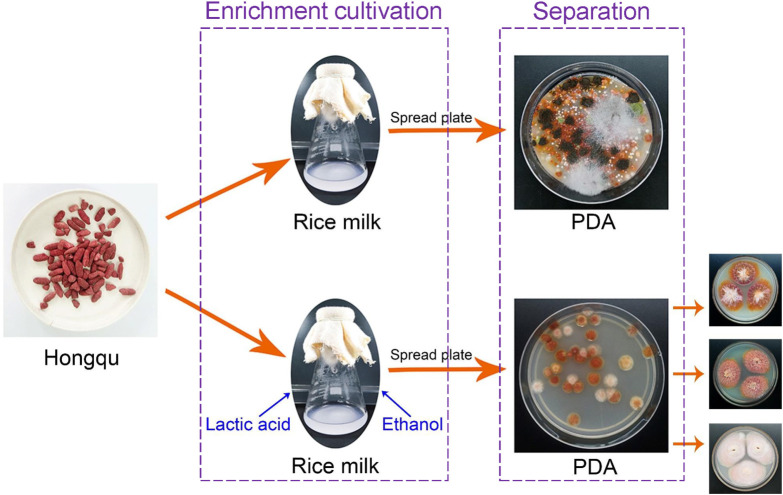

## Introduction

Hongqu, also called red fermented rice, red yeast rice and *Anka*, is produced from steamed rice inoculated with *Monascus* seed culture through solid-state fermentation (Liu et al., 2018). It has been used for thousands of years in Asian countries and is currently used worldwide as a food coloring, fermentation starter and food additives (Tian et al., 2016; Lei et al., 2019). *Monascus*, an essential microorganism for the production of Hongqu, can metabolize a variety of useful enzymes and secondary metabolites (Zhou et al., 2019). For example, liquefying enzymes and saccharifying enzymes contribute to the degradation of starch in grains and the formation of reducing sugars (Shin et al., 2017; Ai et al., 2019); *Monascus* pigment can be used for food coloring and antisepsis (Agboyibor et al., 2018); and lovastatin and γ-aminobutyric acid are beneficial to the development of lipid-lowering and blood pressure-reducing drugs, respectively (Eren et al., 2015; Zhang et al., 2018; Xiong et al., 2019). To fully understand the production and application values of *Monascus*, it is necessary to isolate and purify the original strain from Hongqu to obtain high-quality *Monascus*.

Most Hongqu production follows the manual fermentation techniques of ancient China, so the production process of Hongqu is not strictly controlled (Park et al., 2016; Huang et al., 2019). In addition to *Monascus*, bacteria such as *Lactobacillus* and *Bacillus* and fungi such as yeast, *Aspergillus niger*, and *Aspergillus oryzae* may be present in Hongqu (Lv et al., 2015, 2016). The presence of these microorganisms can cause serious disturbance during the preparation of *Monascus* pure culture from Hongqu. Most of the purification of *Monascus* does not involve exclusive pretreatment and involves direct coating or streaking on mold medium. The media used in these methods include conventional mold media such as potato dextrose agar (Lv et al., 2016), Rose Bengal medium (Huang et al., 2014), malt extract agar (Barbosa et al., 2017) and mold media supplemented with antibiotics or sodium deoxycholate (Zhang et al., 2015). Although these methods are convenient to operate, they cannot exclude the interference of fungi other than *Monascus*. The spores of various microorganisms often diffuse in the culture dish, and repeated operations are required to obtain *Monascus*. Therefore, it is necessary to invent a novel restrictive medium for the enrichment of *Monascus*.

To enrich all kinds of *Monascus* as much as possible, the type and concentration of regulatory factors for purification should meet the following requirements: other microorganisms are eliminated without inactivation of *Monascus*. Thus, *Monascus* must be well tolerated with these regulatory factors. *Monascus* has been reported to have good tolerance characteristics toward lactic acid and ethanol. For example, *Monascus ruber* CBS 127564 can tolerate 14.5% (v/v) lactic acid (Weusthuis et al., 2017), and *Monascus purpureus* FJMR24 can tolerate 18% (v/v) ethanol (Ren et al., 2021). However, although it is not uncommon for microorganisms to tolerate lactic acid or ethanol, just as *Terrilactibacillus laevilacticus* SK5-6 can tolerate approximately 12% (v/v) lactic acid (Prasirtsak et al., 2017) and *Candida tropicalis* can tolerate approximately 30% (v/v) ethanol (Balia et al., 2018), there are very few reports on non-genetically modified microorganisms that can tolerate high concentrations of lactic acid and ethanol simultaneously. *Monascus* has the potential to tolerate lactic acid and ethanol simultaneously, so it is expected to be enriched from Hongqu through a restrictive medium based on the synergistic stress of lactic acid and ethanol.

In this study, we explored the possibility of lactic acid and ethanol as regulatory factors of a restrictive medium for *Monascus* enrichment from Hongqu, and also preliminarily speculated the possible mechanisms on the tolerance of *Monascus* to the synergistic stress of lactic acid and ethanol. These results would help to improve the efficiency of *Monascus* enrichment from Hongqu and provides a reference for better mining of *Monascus* germplasm resources.

## Materials and Methods

### Sources of *Monascus* and Hongqu

*M. serorubescens* A1, *M. lunisporas* A4, *M. sanguineus* B1, *M. kaoliang* B6, *M. argentinensis* B7, *M. purpureus* C1 was isolated from Hongqu by our laboratory (Lv et al., 2012; Cai et al., 2019). Nucleotide sequences for ITS-5.8S rDNA region of these Monascus were deposited in the NCBI database under accession numbers MZ297243-MZ297247 and MZ297277 respectively. Hongqu samples used in this study were collected from different cities in Fujian Province, China: BHQ26 (Hongqu for rice wine brewing, from Ningde city), BHQ33 (Hongqu for rice wine brewing, from Quanzhou city), PHQ30 (Hongqu for pigment production, from Ningde city), WHQ38 (Wuyi Hongqu, a special Hongqu for rice wine brewing that contains more *Aspergillus niger* than other Hongqu, from Fuzhou city).

### Mediums for Microbial Culture and Fermentation

Potato dextrose agar (PDA) (Guangdong Huankai Microbial Sci. & Tech. Co., Ltd., Guangzhou, China) was configured according to the product manual. The modified PDA medium was prepared by adding 0.1 g/L chloramphenicol and 0.5 g/L sodium deoxycholate to the original PDA. The modified potato dextrose water (PD) medium is based on PD medium (Guangdong Huankai Microbial Sci. & Tech. Co., Ltd., Guangzhou, China) with 15 g/L indica rice flour, 10 g/L sodium glutamate, and 5 g/L glycerol. The rice milk containing 15 g/L indica rice flow, 5 g/L glucose and 0.1 g/L chloramphenicol was used as the basic medium, and different contents of hydrochloric acid (HCl), lactic acid and ethanol were added to this rice milk as the restrictive medium according to the experimental requirements.

### Tolerance Test of *Monascus* Against Lactic Acid and Ethanol

All the *Monascus* strains were activated on PDA plates and incubated at 30°C for 7 days under aerobic conditions. Spores were harvested by flooding the surface of the agar with normal saline. The spore suspension was adjusted with sterile saline solution to 10^6^ CFU/mL with a hemocytometer. The *Monascus* spore suspension was connected to different restrictive medium at 1% (v/v) inoculum size and cultured for 7 days. After the fermentation, samples were taken to determine the viable number and biomass increment of *Monascus*.

### Separation of *Monascus* Pure Culture From Hongqu

Hongqu was ground into Hongqu powder by using a sterile mortar. Direct dilution coating method: 0.1500 ± 0.0010 g Hongqu powder was accurately weighed and put into 30 mL sterile saline solution and shake for 90 s. The insoluble particles were filtered out with four layers of gauze and the remaining liquid containing microbial spores diluted and coated on the modified PDA medium. Fermentation-coating method: 0.1500 ± 0.0010 g Hongqu powder was accurately weighed and fermented in a 250 mL shake flask containing 30 mL restrictive medium at 30°C and 200 r/min. After the fermentation, the fermentation broth was diluted and coated on the modified PDA medium for 4 days at 30°C.

### Effects of Lactic Acid and Ethanol on Morphology and Metabolism of Purified *Monascus* Progeny

The spore suspension of *Monascus* A1, A4, B1, B6, B7, C1 were respectively inoculated into the restrictive medium containing 3.98% (v/v) lactic acid and 6.24% (v/v) ethanol for 7 days at 30°C and 200 r/min. The fermentation broth of the restrictive medium was used as the treatment group, and the spore suspension harvested from the PDA plate was used as the control group. Each group contained 6 *Monascus* species. The two groups of *Monascus* have activated to PDA plates again and then spotted on the modified PDA for 7 days at 30°C to observe the colony morphology, at the same time, fermented in a 250 mL shake flask containing 30 mL modified PD medium at 30°C and 200 r/min for 7 days to detect metabolites.

### Metabolic Behaviors of Fungus Isolated From BHQ33 Against Lactic and Ethanol Stress

All the fungus isolated from BHQ33 were activated to PDA plates and incubated at 30°C for 7 days under aerobic conditions. Spores were harvested by flooding the surface of the agar with sterile saline solution and were adjusted with normal saline to 10^6^ CFU/mL with a hemocytometer. The above fungal spore suspensions were first respectively inoculated at 1% (v/v) inoculum size into basal medium (rice milk, containing 15 g/L indica rice flour, 5 g/L glucose, and 0.1 g/L chloramphenicol) for adaptive growth at 30°C and 200 r/min for 7 days, followed by centrifugation at 3,000 r/min for 15 min to collect the cells, which were then washed 3 times with sterile saline solution. The washed cells were resuspended to 10 mg/mL with sterile saline solution and were connected to different selective media at 10% (v/v) inoculum size and cultured for 24 h. There are four kinds of selective media in this part experiment: basal medium without lactate and ethanol (control), basal medium supplemented with 3.98% (v/v) lactic acid (experimental group 1), basal medium supplemented with 6.24% (v/v) ethanol (experimental group 2) and basal medium supplemented with both 3.98% (v/v) lactic acid and 6.24% (v/v) ethanol (experimental group 3).

### Analysis of Fungi Quantity

In the tolerance test of *Monascus*, the plate count and glucosamine content of biomass were used to analyze the biomass increment of *Monascus* in different selective media, and the plate count method was used to determine the number of viable fungi in the samples in other experiments. Glucosamine is the main component of chitin in *Monascus* cell wall, and its content is often used to predict the biomass of *Monascus* in samples containing indica rice. With the dry cell of *Monascus* as a standard reference, the standard curve between the absorbance value and the dry weight of the cell was drawn after four steps of acidolysis, neutralization, Elson-Morgan reaction and colorimetric determination (Sakural et al., 1977; Chysirichote et al., 2013). The sample was converted into biomass based on the standard curve.

### Identification of Isolated Fungal Strains

The fungi isolated and purified from the modified PDA medium were identified by DNA analysis: ITS-5.8S rDNA region was partially amplified and sequenced using primers ITS1/ITS4 as described by Park et al. (2004). Each 25 μL PCR reaction mixture consisted of 1 μL DNA template, 12.5 μL 2 × Power Taq PCR MasterMix (containing MgCl_2_) (Sangon Biotech Co., Ltd. Shanghai, China), 1 μL primer, and ddH_2_O was added to a final volume of 25 μL. The PCR was conducted under the following conditions: initial denaturation at 95°C for 5 min, 35 cycles of denaturation at 94°C for 45 s, annealing at 55°C for 45 s, extension at 72°C for 120 s, followed by a final extension cycle at 72°C for 10 min prior to maintaining the mixture at 4°C. Products were analyzed on 1.5% agarose gel containing 0.1 μL/mL of 4S Red Plus nucleic acid stain (Sangon Biotech Co., Ltd. Shanghai, China) and visualized under UV light (UV source GelDoc 1000, Bio-Rad). PCR products were gel-purified with GFX^TM^ PCR DNA and Gel Band Purification Kit (Amersham Biosciences AB, Uppsala), according to the manufacturer’s instructions. Purified PCR products were directly sequenced using the ABI prism 3730 DNA analyzer (Applied Biosystems, Foster). Sequences were analyzed using Blast at NCBI.^1^ Nucleotide sequences for ITS-5.8S rDNA region of isolates represent *Monascus purpureus* BHQ33.M01, *Monascus purpureus* BHQ33.M02, *Monascus purpureus* BHQ33.M03, *Saccharomyces cerevisiae* BHQ33.S01, *Aspergillus niger* BHQ33.AN01, *Lichtheimia ramosa* BHQ33.L01, *Trichoderma* BHQ33.T01, and *Aspergillus flavus* BHQ33.AF01 were deposited in the NCBI database under accession numbers MW581230–MW581237.

### Analysis of Metabolites in the Regenerated Progenies of *Monascus*

The analytical method of γ-aminobutyric acid was referenced to the agricultural industry-standard NY/T 2890-2016 of the people’s Republic of China, unit: μg/mL. The pigment, lovastatin and citrinin were extracted with 70% (v/v) ethanol at 60°C for 2 h. The mycelium of the sample was removed by centrifugation at 5,000 r/min for 10 min, and the supernatant was diluted and filtered through a 0.22 μm filter membrane to be tested. The content of *Monascus* pigment was predicted by the absorbance value using a spectrophotometer (U1900, Hitachi, Japan) at 505 nm referred to the national standards of the people’s Republic of China GB 1886.19-2015, unit: U/mL; The HPLC (L2000, Hitachi, Japan) analysis method of lovastatin referred to the light industry standard of the people’s Republic of China GB/T 2847-2007, unit: μg/mL; The HPLC analysis method of citrinin refers to the national standard of the people’s Republic of China GB 5009.222-2016, unit: ng/mL.

The amylase activity was measured using an α-amylase assay kit (all kits used in the current study were purchased from Nanjing Jiancheng Bioengineering Institute, Nanjing, China, unless otherwise indicated) based on QB/T 4257-2011. One unit of amylase activity was defined as the quantity of enzyme metabolized by 1 g mycelium that was required to hydrolyze 1 mg soluble starch per hour, at 35°C and pH 4.6. The glucoamylase activity was measured according to the Light Industry Standard of the People’s Republic of China QB/T 4257-2011 with slight modifications. One unit of glucoamylase was defined as the amount of enzyme metabolized by 1 g mycelia that was required to release 1 mg glucose per hour from soluble starch, at 35°C and pH 4.6.

### Metabolic Behaviors of Fungus Isolated From BHQ33 Against Lactic Acid and Ethanol Stress

The fermented medium was diluted to 10 mg/mL with sterile saline solution. 20 mL aliquot of the sample was centrifuged at 3,000 r/min for 15 min, and the supernatant, which had been filtered through a 0.45 μm filter, was used as a sample to be tested for extracellular metabolites. The centrifuged cells were washed 3 times with sterile saline solution and resuspended to 15 mL, followed by ultrasonic disruption (FS-200T, SXSONIC, China) at 200 W for 30 min under ice bath conditions. The ultrasonic fragmentation cycle times were as follows: 5 s working, 5 s stagnating, and 20 kHz frequency. After disruption, cell debris was removed by centrifugation at 4,500 r/min for 20 min, and the supernatant was used as the sample to be tested for intracellular metabolites.

The analytical methods for lactic acid and ethanol were referred to previous studies by Jiang et al. (2019), and Cai et al. (2019), respectively. The reducing sugars were determined by 3, 5-dinitrosalicylicacid method (Xu et al., 2014). Protein concentrations were measured using a coomassie (Bradford) protein assay kit. Soluble starch content and amylase activity were measured using an α-amylase assay kit with some modifications. In detail, soluble starch content is measured by the chromogenic reaction of the iodide solution with the extracellular solution, and the definition of one unit of amylase activity in this system was modified as the quantity of enzyme metabolized by 1 mL extracellular solution that was required to hydrolyze 1 mg soluble starch per hour, at 35°C and pH 4.6. Total superoxide dismutase (SOD), malondialdehyde (MDA), catalase (CAT), and total glutathione peroxidase (GSH-Px) levels of intracellular metabolism were measured with the assay kits according to the manufacturer’s instructions (Nanjing Jiancheng Institute of Biotechnology, China).

### Statistical Analysis

The experiments were conducted in triplicate, and the results were presented as the mean ± standard deviation (SD). One-way analysis of variance (ANOVA) was performed across multiple groups to test significance (*p* < 0.05) using SPSS 23.0 software (SPSS Inc., Chicago, United States). A histogram was generated using Origin 2018 software (OriginLab, Northampton, MA, United States). Response surface design (RSD) was carried out by Design-Expert V8.0.6.1software (Stat-Ease, Inc., Minneapolis, MN, United States).

## Results

### Tolerance of *Monascus* to Lactic Acid and Ethanol

**FIGURE 1:**
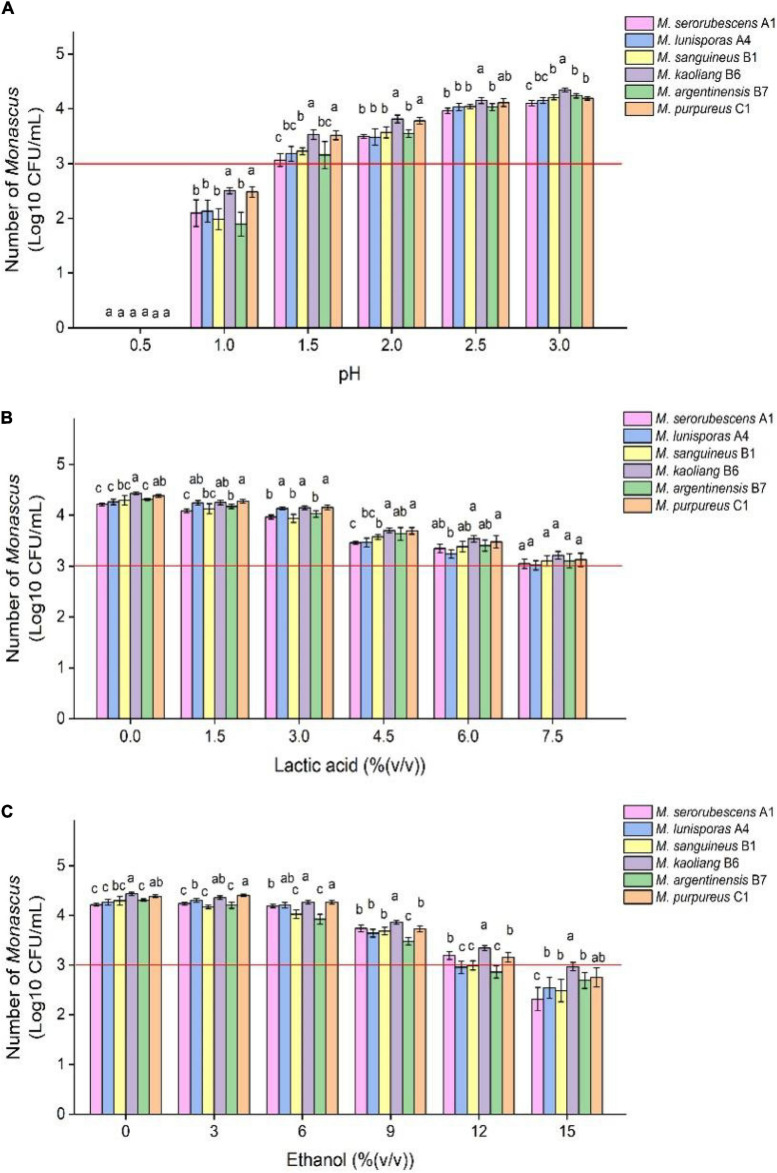
Tolerance test of different species of *Monascus* to different growth inhibitors. The six *Monascus* species were cultured in restrictive medium containing different concentrations of HCl **(A)**, lactic acid **(B)** and ethanol **(C)** at 30°C and 200 r/min for 7 days, and the number of viable spores of *Monascus* was determined by plate coating after fermentation. Error bars indicate the standard deviation (SD) of the means (*n* = 3), and different lowercase letters represent significant differences in the number of viable cells of different *Monascus* strains under the same treatment condition (*p* < 0.05). The red auxiliary line indicates that the initial amount of *Monascus* is 1.0 × 10^3^ CFU/mL.

The range of common tolerance of *Monascus* to growth regulatory factors can be roughly determined by tolerance tests with multiple species *Monascus*, which facilitates selection of the type and concentrations of growth regulatory factors added to the restrictive medium. *Monascus* can grow in the medium containing low concentrations of lactic acid and ethanol with a certain number of live spores (Figure 1) and mycelium (Figure 1 and Supplementary Figure 1). Specific concentrations of lactic acid [7.5% (v/v)] inhibited but did not eliminate the growth of *Monascus* because the number of viable spores in the fermentation broth did not change much compared with the initial amount, and a similar result was observed when ethanol was added to 12.0% (v/v). The inhibition of spore activity of *Monascus* by 7.5% (v/v) lactic acid did not depend exclusively on H^+^ concentration because the pH of this medium was 2.1 (Supplementary Figure 2), and *Monascus* was found to tolerate 10^–1.5^ mol/L HCl (pH = 1.5).

**FIGURE 2:**
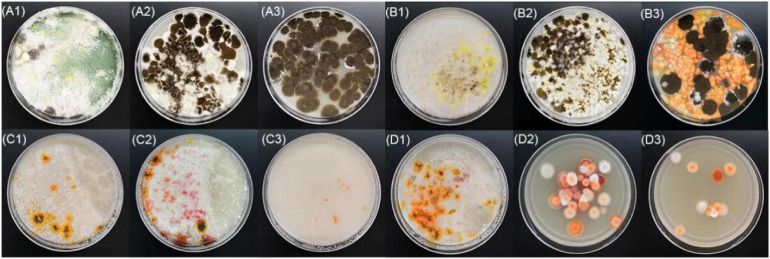
Effect of different growth inhibitors on the enrichment of *Monascus* from Hongqu BHQ33. Hongqu BHQ33 was added to ferment respectively with different fermentation media containing 10^− 2.5^mol/L HCl **(A1)**, 10^− 2^mol/L HCl **(A2)**, 10^− 1.5^mol/L HCl **(A3)**, 1.5% (v/v) lactic acid **(B1)**, 3% (v/v) lactic acid **(B2)**, 4.5% (v/v) lactic acid **(B3)**, 3% (v/v) ethanol **(C1)**, 6% (v/v) ethanol **(C2)**, 9% (v/v) ethanol **(C3)**, 1.5% (v/v) lactic acid & 3% (v/v) ethanol **(D1)**, 3% (v/v) lactic acid & 6% (v/v) ethanol **(D2)**, 4.5% (v/v) lactic acid & 9% (v/v) ethanol **(D3)** at 30°C and 200 r/min for 7 days, and then coated on the modified PDA medium with the fermentation broth to culture for 4 days at 30°C.

BHQ33 Hongqu powder was added to the medium containing different growth regulatory factors for 7 days (Figure 2). After fermentation, the fermentation broth was diluted and coated in modified PDA for observation. Coating results showed that adding HCl or ethanol alone did not help to obtain *Monascus*, while adding 4.5% (v/v) lactic acid caused *Monascus* to appear in the plate. However, lactic acid alone did not inhibit *Aspergillus niger*, which grows significantly faster than *Monascus*, a situation that was unfavorable for obtaining as many different kinds of *Monascus* as possible. Combined with the results of Figures 2B3,C3, 9% (v/v) ethanol was able to eliminate *Aspergillus niger*, while 4.5% (v/v) lactic acid could eliminate yeast, and adding lactic acid and ethanol simultaneously was able to enrich *Monascus* (Figures 2D2,D3).

### Optimization of Lactic Acid and Ethanol Addition in the Restrictive Medium

**FIGURE 3:**
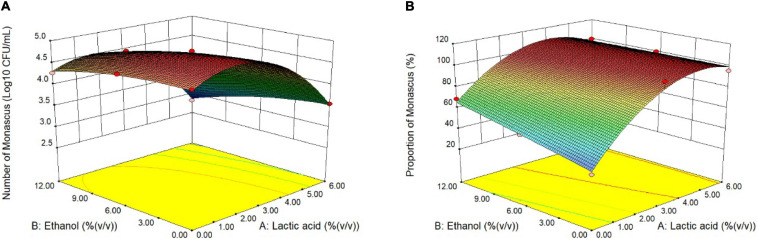
Response surface optimization of lactic acid and ethanol addition in restrictive medium. Hongqu BHQ33 was added to the restrictive medium containing different concentrations of lactic acid and ethanol for fermentation at 30°C and 200 r/min for 7 days, the number of viable *Monascus* spores **(A)** and the proportion of the number of *Monascus* **(B)** were calculated after the fermentation broth was diluted and spread on the modified PDA medium to culture for 4 days at 30°C.

To eliminate fungi other than *Monascus* from BHQ33 Hongqu powder and to enrich as much *Monascus* as possible, response surface optimization was applied to optimize the amount of lactic acid and ethanol added to the restrictive medium. According to the tolerance test of *Monascus* in Figure 1, the concentration ranges of lactic acid and ethanol in the restrictive medium were selected to be 0∼6% (v/v) and 0∼12% (v/v), respectively. The response surface methodology uses a 2-factor 3-level model of Design-Expert software. The test scheme and results are listed in Table 1 and Figure 3. The test results in Table 1 confirm the conclusion in Figures 2C,D that it is difficult to grow *Monascus* only in culture dishes by using single lactic acid or ethanol. The fitting equation of the response surface model is as follows:

**TABLE 1:** Response surface test scheme and results for optimizing the addition of lactic acid and ethanol.

Number	Lactic acid(%(v/v))	Ethanol (%(v/v))	The viable count of *Monascus* (Log10 CFU/mL)	The proportion of *Monascus* (%)
1	3	6	4.758	100
2	0	12	4.271	68.77
3	3	12	4.448	100
4	0	0	4.668	36.42
5	3	6	4.771	100
6	6	12	2.824	100
7	6	0	3.551	95.36
8	3	6	4.678	100
9	0	6	4.609	52.47
10	3	0	4.822	89.95
11	3	6	4.655	100
12	3	6	4.671	100
13	6	6	3.368	100

*Three parallel experiments were conducted in each group. For the convenience of software operation, only the mean value is input.*

(1) $$\begin{array}{c} Y_{1}=4.64507+0.32330A+0.01023B-0.00458AB-0.08453A^{2}-0.00317B^{2} \end{array}$$

(2) $$\begin{array}{c} Y_{2}=36.74284+24.53848A+3.50424B-0.38486AB-2.42989A^{2}-0.086916B^{2} \end{array}$$

where *Y*_1_ is the number of *Monascus* colonies, unit: Log10 CFU/mL; *Y*_2_ is the proportion of *Monascus*, unit: %; *A* is the amount of lactic acid added, unit: % (v/v); and *B* is the amount of ethanol added, unit: % (v/v). The constraints of equations (1) and (2) are *f*(*A*) = min{*A*}, *f*(*B*) = min{*B*}, *f*(*Y*_1_) = man{*Y*_1_}, *f*(*Y*_2_) = man{*Y*_2_}, 0≤A≤6.0, 0≤B≤12.0 and *Y*_2_≥100. The optimal solution is A = 3.98, B = 6.24, *Y*_1_ = 4.420, and *Y*_2_ = 104.8 (according to the actual situation, *Y*_2_ = 100).

Therefore, when the amount of lactic acid and ethanol reached 3.98% (v/v) and 6.24% (v/v), respectively, the amount of *Monascus* was 4.420 Log10 CFU/mL, and the proportion of *Monascus* was 100%. The actual plate coating verification shows that the number of viable *Monascus* under this condition is 4.49 ± 0.12 Log10 CFU/mL, accounting for 100% (3 strains of fungi could be obtained from the plate (Supplementary Figure 3), and all were *Monascus purpureus* after ITS gene sequencing).

### Dynamics of *Monascus* in Restrictive Medium During the Fermentation Process

**FIGURE 4:**
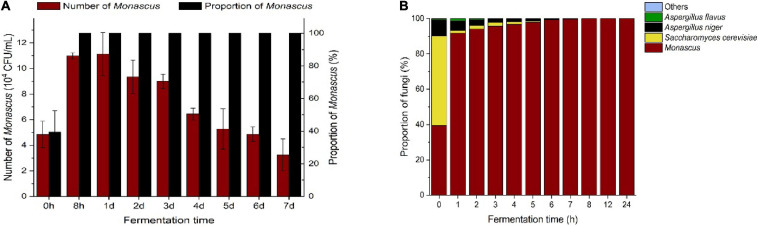
Trends of *Monascus* and other culturable dominant fungi in the optimized restrictive medium supplemented with Hongqu BHQ33 during the fermentation process. Hongqu BHQ33 was added to the optimized restrictive medium containing 3.98% (v/v) lactic acid and 6.24%(v/v) ethanol for fermentation at 30°C and 200 r/min for 7 days, the number of *Monascus* and its proportion over time **(A)** and proportion of culturable dominant fungi over time **(B)** were calculated after the fermentation broth was diluted and spread on the modified PDA medium to culture for 4 days at 30°C. Error bars indicate the standard deviation (SD) of the means (*n* = 3).

After the optimal addition of lactic acid and ethanol in the restrictive medium, the number and proportion of *Monascus* in the fermentation broth containing BHQ33 powder rapidly increased and gradually decreased from the first day to the 7th day of fermentation (Figure 4A). However, the samples fermented to the 7th day did not affect the enrichment of *Monascus*, and the amount of *Monascus* still exceeded 1.0 × 10^4^ CFU/mL, accounting for 100%. To study the changing pattern of flora changes in this culture system more meticulously and determine the optimal enrichment duration, the enrichment process from 0 to 24 h was followed up by sampling and spread analysis (Figure 4B). The dominant fungi in the fermentation broth were *Monascus*, *Saccharomyces cerevisiae*, *Aspergillus niger* and *Aspergillus flavus*, while *Lichtheimia ramos* and *Trichoderma* were observed sporadically. From the initial state to 8 h of enrichment culture, the proportion of fungi except for *Monascus* gradually decreased until they disappeared. To obtain *Monascus* quickly and conveniently, sampling and coating are were carried out between 8 and 24 h after enrichment culture, which can save time and eliminate the interference of other fungi.

### Metabolic Behaviors of *Monascus* Against Lactic Acid and Ethanol Stress

**FIGURE 5:**
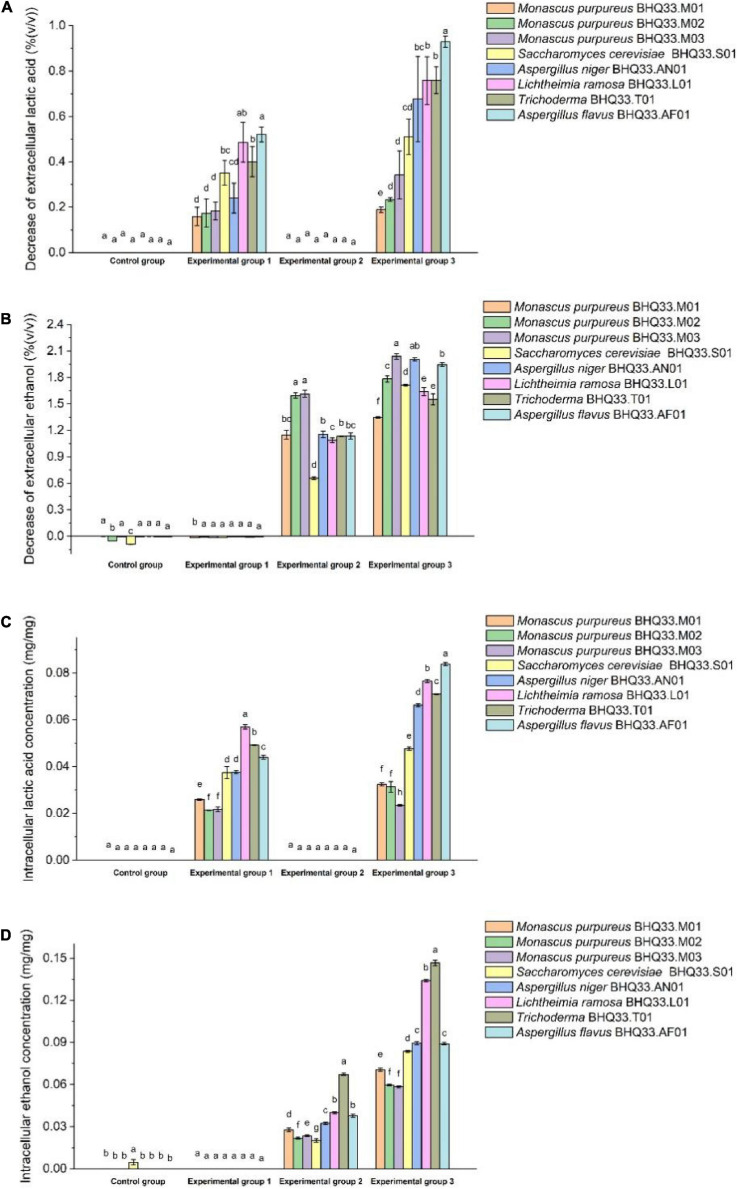
Changes in intracellular and extracellular lactic acid and ethanol contents before and after fermentation by different fungi isolated from Hongqu BHQ33. Different fungi isolated from Hongqu BHQ33 were cultured in medium containing different concentrations of lactic acid and ethanol at 30°C and 200 r/min for 24 h, and the decrease of extracellular lactic acid **(A)**, the decrease of extracellular ethanol **(B)**, the accumulation of intracellular lactic acid **(C)** and the accumulation of intracellular ethanol **(D)** were detected. In the initial medium, control group was basal medium without lactate and ethanol, experimental group 1 was supplemented with 3.98% (v/v) lactic acid in basal medium, experimental group 2 with 6.24% (v/v) ethanol, and experimental group 3 with both 3.98% (v/v) lactic acid and 6.24% (v/v) ethanol. Error bars indicate the standard deviation (SD) of the means (*n* = 3), and different lowercase letters represent significant differences in the detection indexes of fungi isolated from Hongqu BHQ33 under the same treatment conditions (*p* < 0.05).

The fungi isolated from BHQ33 were inoculated into the restrictive medium containing different concentrations of lactic acid and ethanol for 24 h and sampled for analysis. Compared with experimental groups 1 and 2, the decreases in extracellular lactate and ethanol in experimental group 3 were significantly increased (Figures 5A,B), indicating that the cell membrane permeability could be changed by the synergistic effect of lactate and ethanol. Under the simultaneous stress of lactic acid and ethanol, *Monascus* reduced the amount of lactic acid in the cell, and the intracellular scavenging ability of lactic acid and ethanol was better than that of other fungi (Figures 5C,D).

**FIGURE 6:**
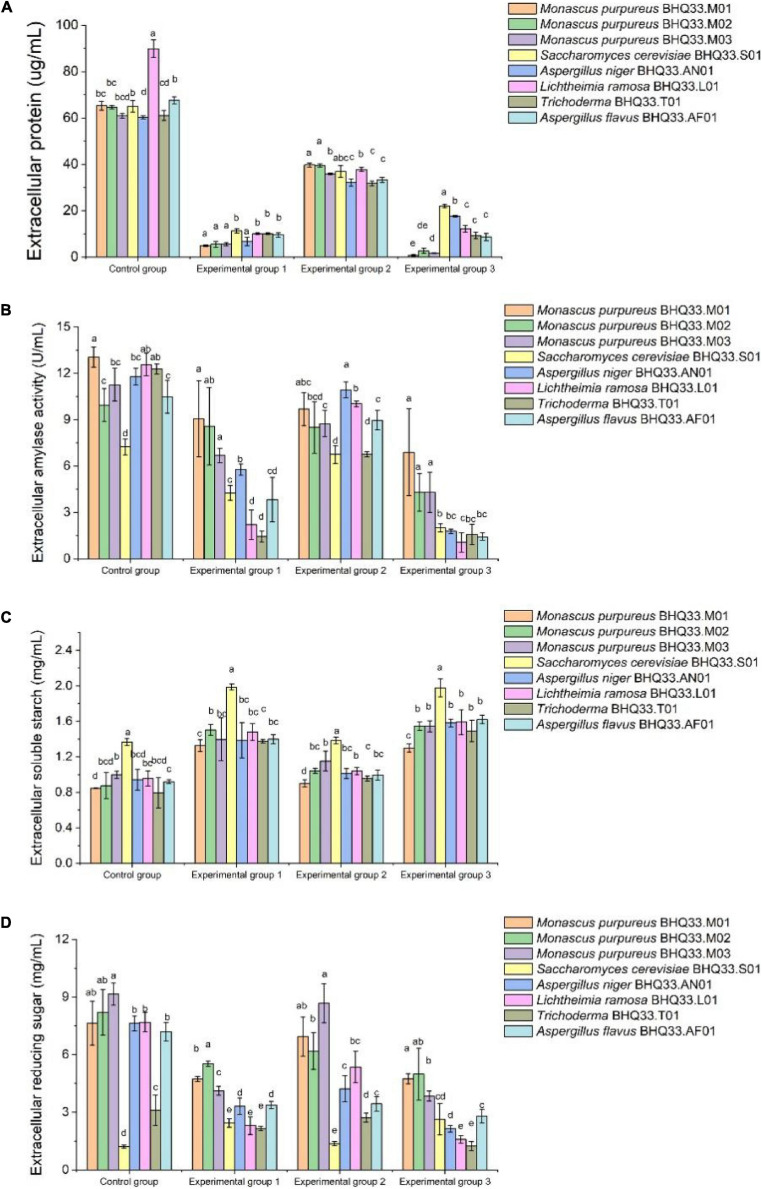
Degradation and utilization of substrates by different fungi isolated from Hongqu BHQ33. Different fungi isolated from Hongqu BHQ33 were cultured in medium containing different concentrations of lactic acid and ethanol at 30°C and 200 r/min for 24 h, and extracellular protein concentration **(A)**, extracellular amylase activity **(B)**, extracellular soluble starch concentration **(C)**, and extracellular reducing sugar concentration **(D)** were detected. In the initial medium, control group was basal medium without lactate and ethanol, experimental group 1 was supplemented with 3.98% (v/v) lactic acid in basal medium, experimental group 2 with 6.24% (v/v) ethanol, and experimental group 3 with both 3.98% (v/v) lactic acid and 6.24% (v/v) ethanol. Error bars indicate the standard deviation (SD) of the means (*n* = 3), and different lowercase letters represent significant differences in the detection indexes of fungi isolated from Hongqu BHQ33 under the same treatment conditions (*p* < 0.05).

Extracellular metabolites were analyzed (Figure 6). For the experimental groups supplemented with lactic acid (groups 1 and 3), the amylase activity and extracellular reducing sugar concentration of *Monascus* were higher than those of other fungi, and their extracellular soluble starch contents and those of other filamentous fungi were not significantly different. Extracellular amylase can peel soluble starch from rice flour granules and then degrade soluble starch into reducing sugars, so it can be speculated that the liquefaction capacity and saccharification capacity of *Monascus* under lactic acid stress are better than those of other fungi, while higher contents of reducing sugars can provide more energy sources for cells.

**FIGURE 7:**
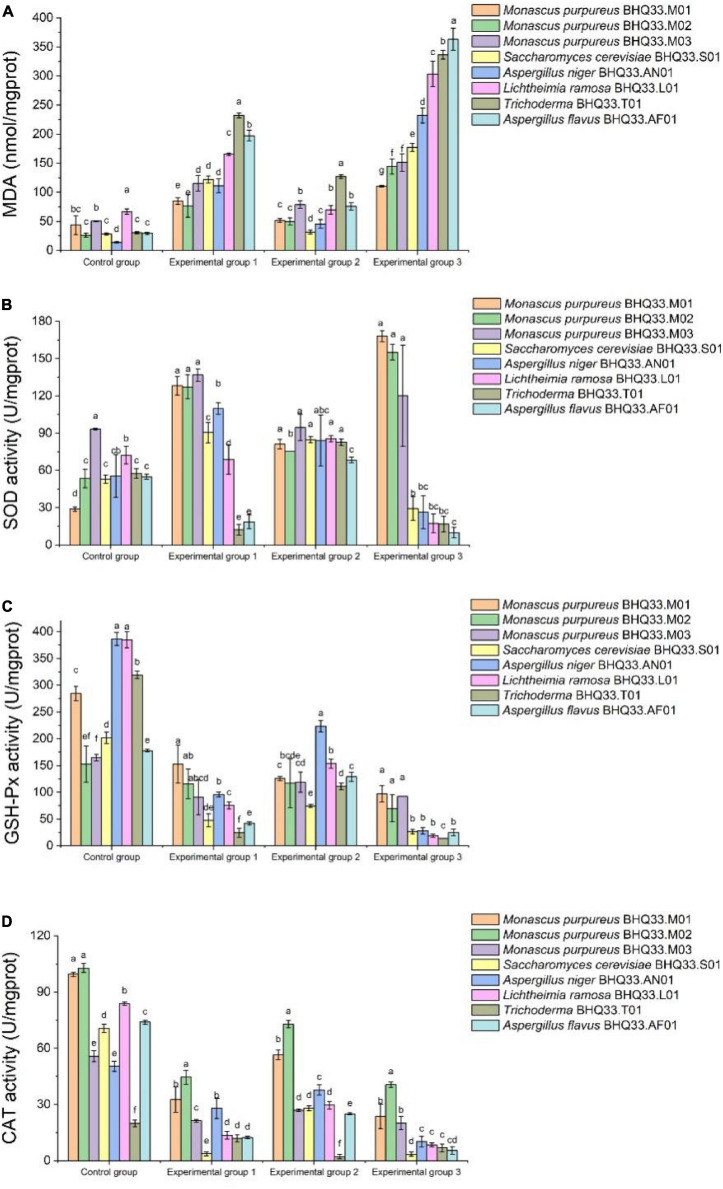
Intracellular antioxidant capacity of different fungi isolated from Hongqu BHQ33 under lactic and ethanol stress. Different fungi isolated from Hongqu BHQ33 were cultured in medium containing different concentrations of lactic acid and ethanol at 30°C and 200 r/min for 24 h, and intracellular metabolites malondialdehyde (MDA) **(A)**, total superoxide dismutase (SOD) **(B)**, total glutathione peroxidase (GSH-Px) **(C)**, and catalase (CAT), **(D)** were detected. In the initial medium, control group was basal medium without lactate and ethanol, experimental group 1 was supplemented with 3.98% (v/v) lactic acid in basal medium, experimental group 2 with 6.24% (v/v) ethanol, and experimental group 3 with both 3.98% (v/v) lactic acid and 6.24% (v/v) ethanol. Error bars indicate the standard deviation (SD) of the means (*n* = 3), and different lowercase letters represent significant differences in the detection indexes of fungi isolated from Hongqu BHQ33 under the same treatment conditions (*p* < 0.05).

Intracellular metabolites were analyzed (Figure 7). Lactic acid and ethanol can cause lipid peroxidation damage of cell membrane, so the content of MDA in experimental group 1 and experimental group 2 is higher than that in the control group. Lipid peroxidative damage to the cell membrane was more intense when lactic acid and ethanol were present simultaneously (Figure 7A). Under the synergistic stress of lactate and ethanol, the accumulated amount of MDA in *Monascus* cells was significantly lower than that in other fungi, which might be related to the higher activities of intracellular antioxidant enzymes (SOD, GSH and CAT). This antioxidant enzyme is able to scavenge oxygen free radicals and reduce the oxidative damage of polyunsaturated fatty acids in biofilms, which has a positive effect on maintaining the structure and function of biofilms.

## Discussion

In addition to a large number of *Monascus*, Hongqu also contains lactic acid bacteria, yeasts, and others (Liu et al., 2018; Hong et al., 2020). In the process of applying Hongqu to brew rice wine, it was found that *Monascus* has a certain resistance to lactic acid and alcohol (He et al., 2016; Cai et al., 2019; Liang et al., 2020), while the resistance of other microorganisms in the system is different from it (Lv et al., 2015; Huang et al., 2018). We speculate that it’s a potential way to screen *Manascus* using the synergistic effect of lactic acid and ethanol, so finding the right ratio and concentration of both is the key point. In this study, when 4.5% (v/v) lactic acid was added alone, *Monascus* and *Aspergillus niger* coexisted; while 9% (v/v) ethanol was added alone, *Monascus* and *Saccharomyces cerevisiae* coexisted. After further optimization by response surface method, *Monascus* could be successfully enriched only when 3.98% (v/v) lactic acid and 6.24% (v/v) ethanol were added to the enrichment medium simultaneously. Traditionally, growth regulatory factors have been used to screen microbial strains, but they also may lead to the targeted domestication of the target strain, resulting in the loss of some metabolic functions (Yang et al., 2018). Fortunately, the novel restrictive medium for *Monascus* enrichment based on the synergistic stress of lactic acid and ethanol can shorten the pretreatment time without repeated operations, avoiding the abnormal changes in the metabolic characteristics of *Monascus* progeny to some extent. The morphological characteristics and metabolic function of *Monascus* progeny were not significantly changed after the treatment with the developed restrictive medium based on the synergistic stress of lactic acid and ethanol (Supplementary Figure 4 and Supplementary Table 1).

**FIGURE 8:**
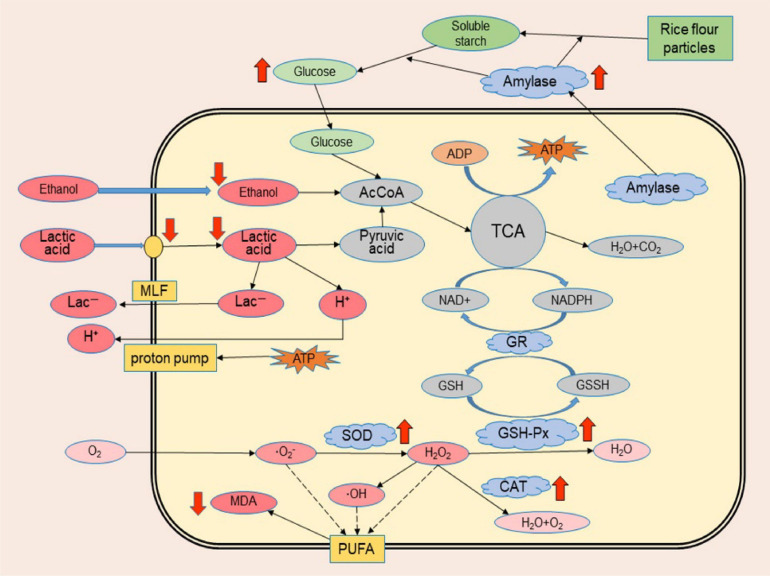
Schematic illustration of the possible tolerance mechanism of *Monascus* to synergistic stress of lactic acid and ethanol. Red arrows indicate significant metabolic differences between *Monascus* and other fungi isolated from BHQ33 in the optimized restrictive medium containing both lactic acid and ethanol, with arrows up-regulated and arrows down-regulated.

When cells are stimulated or stressed, the metabolic activities related to environmental adaptation become active, especially those related to substance transport, substance transformation, and energy metabolism processes. According to previous studies, lactic acid bacteria can resist the damage of acid to cells through neutralization process, biofilm and cell density, proton pump, protection of macromolecules, preadaptation and cross-protection, and effect of solutes (Wang et al., 2018); yeast can resist the damage of ethanol to cells through modification of the membrane, amino acids, trehalose and vacuolar proton-translocating ATPase functions (Saini et al., 2018). However, the tolerance mechanism of *Monascus* to the synergistic stress of lactic acid and ethanol has not been reported. The cell membrane is the key site to defend against environmental growth inhibitors, while the protection mechanism of the cell membrane is related to the kind and proportion of unsaturated fatty acids on the membrane because these fatty acids directly affect the fluidity of the phospholipid bilayer and the running capacity of carrier proteins on the membrane (Li et al., 2011; Fonseca et al., 2019; Liu et al., 2020). For example, the addition of oleic acid to lipid-poor media significantly reduced oxidative damage to *Saccharomyces cerevisiae* cell membranes and resulted in improved resistance to oxidative stress (Landolfo et al., 2010). In addition, lactic acid and ethanol can cause oxidative damage to cells. Excessive reactive oxygen species will attack unsaturated fatty acids on the cell membrane, resulting in increased intracellular MDA, which may cause cross-linking polymerization of life macromolecules such as proteins and nucleic acids and have a toxic effect on cells (Talbi et al., 2019; Požgajová et al., 2020). The oxidative damage of unsaturated fatty acids also affects the resistance of the cell membrane to external growth inhibitors (Fonseca et al., 2019; Liu et al., 2020). *Monascus* has an active system of fatty acid metabolism (Peters et al., 1993; Moharram et al., 2012). The polyketide synthesis (pigment, lovastatin and citrinin) of *Monascus* are associated with fatty acid metabolism which also provides unsaturated long-chain fatty acids to serve as synthetic feedstocks for phospholipids on biological membranes (Wang, 2006; Panda and Ali, 2012; Balakrishnan et al., 2014; Thomanek et al., 2018). Therefore, it can be speculated that the simultaneous tolerance of *Monascus* to lactic acid and ethanol may be associated with the protective function of cell membranes, and this series of metabolic processes is involved in maintaining the structure and function of cell membranes. In the presence of higher concentrations of lactic acid and ethanol simultaneously, *Monascus* is more resistant to cell invasion by lactic acid than other fungi and alleviates the interference of lactic acid and ethanol on the normal metabolism of cells; intracellular antioxidant enzymes are able to reduce the oxidative damage of unsaturated fatty acids on the cell membrane; and higher activity of extracellular amylase and higher concentrations of extracellular reducing sugars are able to provide energy for the cell to defend against stress (Figure 8).

It is worth mentioning that an attempt to prejudge by high-throughput sequencing how many kinds of *Monascus* species are present in the Hongqu BHQ33 revealed only *Monascus purpureus* in this sample (Supplementary Figure 5), but it is not clear how many different types of *Monascus* exist. This study obtained 3 strains of *Monascus purpureus* with different morphological types by using optimized restricted medium supplemented with lactic acid and ethanol (Supplementary Figure 3), which illustrated that a suitable culture method was able to increase the discrimination ability of *Monascus* species and subspecies levels and could also assist in analyzing the species distribution of *Monascus* in Hongqu at the species level.

**FIGURE 9:**
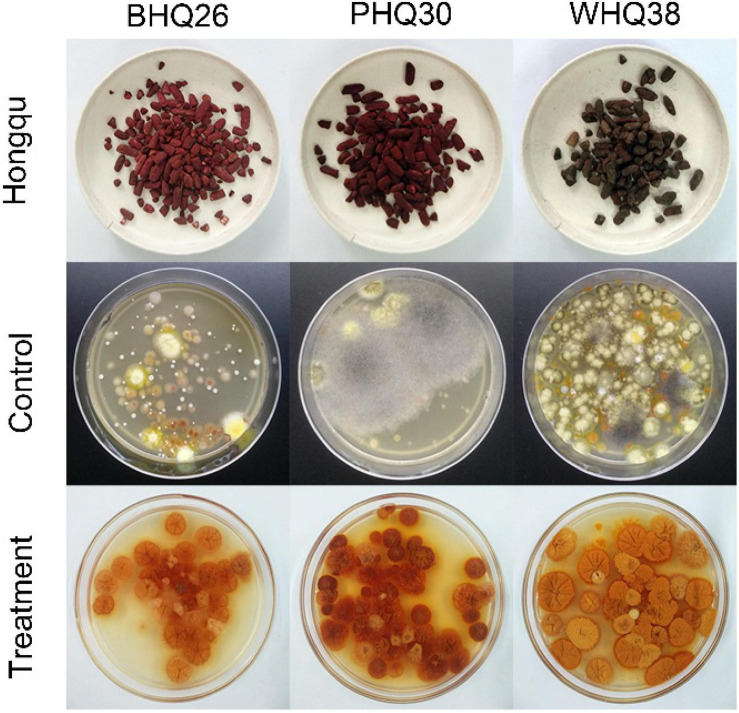
Application of the optimized restricted medium for enrichment of *Monascus* from different Hongqu. The direct dilution coating method (control group) and the fermentation-coating method (treatment group) were used to separate *Monascus* from three Hongqu, respectively. Among them, BHQ26 is a Hongqu for rice wine brewing, PHQ33 is a Hongqu for pigment production, WHQ38 is a special Hongqu for rice wine brewing that contains more *Aspergillus niger* than other Hongqu.

Finally, this method was applied to *Monascus* enrichment from different types of Hongqu (Figure 9). Although the fungi in these Hongqu are very complex and diverse, *Monascus* pure culture can still be enriched and obtained by the restrictive medium based on the synergistic stress of lactic acid and ethanol. The feasibility of lactic acid and ethanol as regulators for directional enrichment of *Monascus* was verified. The possible mechanisms on the tolerance of *Monascus* to the synergistic stress of lactic acid and ethanol can be further explored by transcriptomics and metabolomics, which has important practical significance for the application of *Monascus* in fermentation.

## Conclusion

The preparation of *Monascus* pure culture from Hongqu is easily interfered by other microorganisms. Therefore, the development of restrictive medium for *Monascus* enrichment from Hongqu is of great significance for the screening of excellent *Monascus* strains. This study showed that *Monascus* has good tolerance to lactic acid and ethanol, and retains the vitality of spore germination at high concentrations of lactic acid and ethanol. Under the synergistic stress of lactic acid and ethanol, *Monascus* pure culture can be directionally enriched from Hongqu at appropriate concentrations without affecting the morphological characteristics and metabolic function in *Monascus* progeny. *Monascus* can maintain the barrier function of of its cell membrane and help it defend against the synergistic stress of lactic acid and ethanol. The novel restrictive medium developed for *Monascus* enrichment from Hongqu based on the synergistic stress of lactic acid and ethanol is of great value for the construction of *Monascus* strain libraries and better development of their germplasm resources.

## Data Availability Statement

The datasets presented in this study can be found in online repositories. The names of the repository/repositories and accession number(s) can be found in the article/Supplementary Material.

## Author Contributions

KZ, XL, and LN designed the experiments. KZ, LW, GC, and XZ carried out the experiments. KZ, WZ, and CZ analyzed the experimental results. KZ, XL, LN, ZL, and PR wrote the manuscript. All authors contributed to the article and approved the submitted version.

## Conflict of Interest

The authors declare that the research was conducted in the absence of any commercial or financial relationships that could be construed as a potential conflict of interest.

## Footnotes

1 http://www.ncbi.nlm.nih.gov/
