# Supplementary material for: Development of a Novel Restrictive Medium for Monascus Enrichment From Hongqu Based on the Synergistic Stress of Lactic Acid and Ethanol
Source: Front Microbiol. 2021 Jun 21;12:702951. doi: 10.3389/fmicb.2021.702951 (PMC8256164; doi:10.3389/fmicb.2021.702951)
Supplement: Supplementary file 1 [file Data_Sheet_1.pdf]

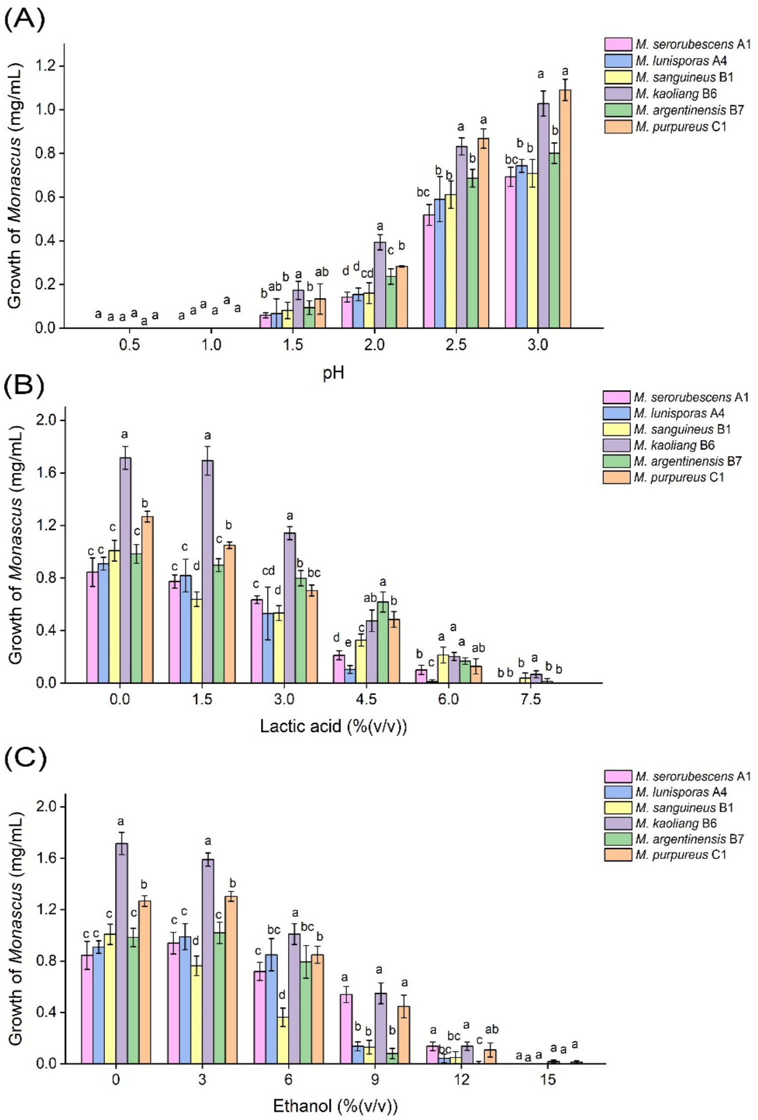

Supplementary Figure S1 Tolerance test of different species of *Monascus* to different growth inhibitors. The tolerance of six *Monascus* species in restrictive media containing different concentrations of pH (A), lactic acid (B) and ethanol (C) was analyzed by determination of biomass increment by glucosamine assay. Error bars indicate the standard deviation (SD) of the means (n=3), and different lowercase letters represent significant differences in biomass of different *Monascus* species under the same treatment conditions ($p < 0.05$).

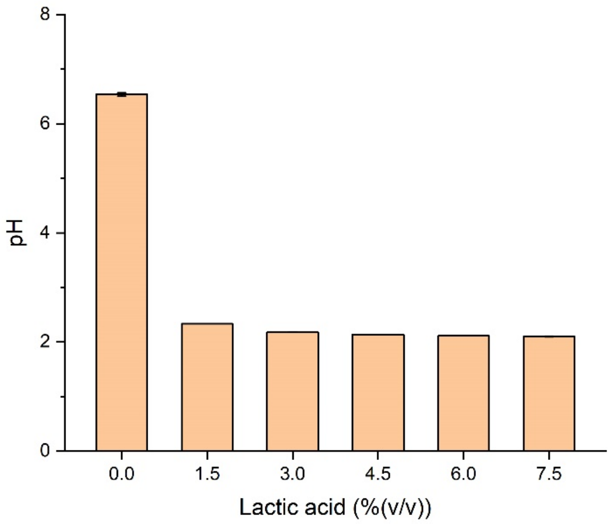

Supplementary Figrue S2 Relationship between lactic acid concentration and pH in initial rice milk medium.

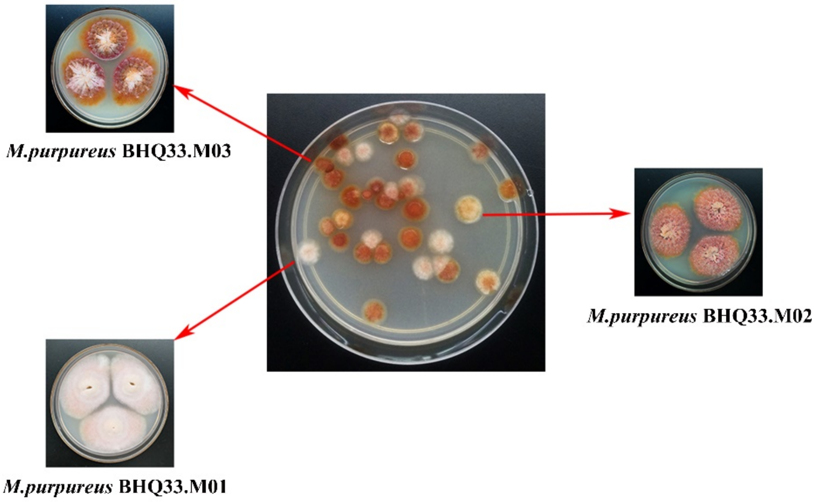

Supplementary Figrue S3 Hongqu BHQ33 powder was added to optimized restrictive media with 3.98% (v/v) lactic acid and 6.24%(v/v) ethanol for 7d at 30℃, 200 r/min and spread on PDA plates to obtain *Monascus*.

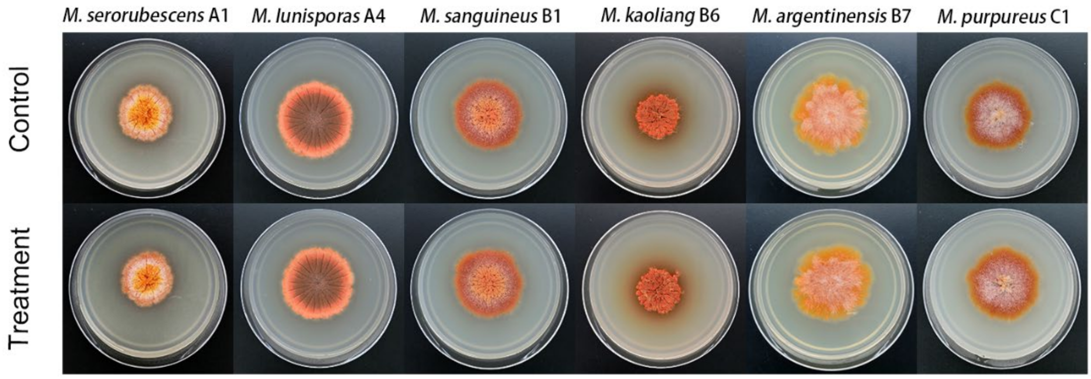

Supplementary Figure S4 Effect of lactic acid and ethanol on morphology after reactivation of *Monascus*. The spore suspension of *Monascus* A1, A4, B1, B6, B7, C1 were inoculated into the selective medium containing 3.98% (v/v) lactic acid and 6.24% (v/v) ethanol for 7d at 30 ℃ and 200 r/min. The fermentation broth of the selective medium was used as the treatment group, and the spore suspension harvested from the PDA plate was used as the control group. Each group contained 6 *Monascus* species. The two groups of *Monascus* have activated to PDA plates again and then spotted on the modified PDA for 7d at 30℃ to observe the colony morphology.

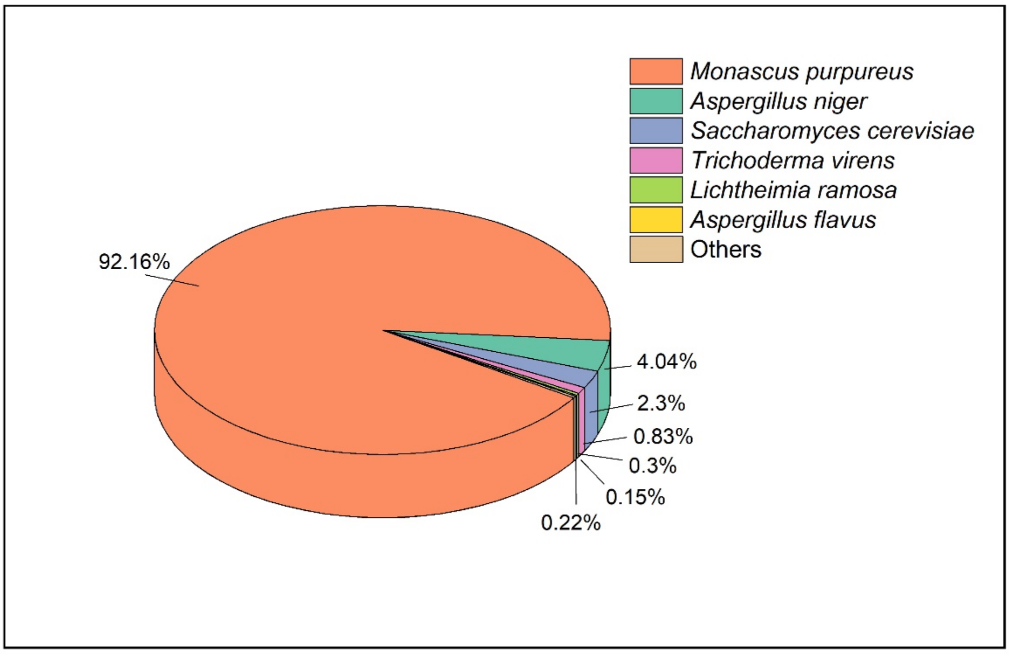

Supplementary Figure S5 High throughput sequencing of fungi of Hongqu BHQ33. *Monascus purpureus* accounted for 100% of *Monascus*. Other fungi not listed include *Candida maltosa*, *Candida tropicalis*, *Mortierella kuhlmanii*, *Cyberlindnera fabianii*, *Meyerozyma caribbica*, *Mortierella humilis*, *Mortierella kuhlmanii*, *Talaromyces radicus* and unclassified.

Supplementary Table S1 Effect of lactic acid and ethanol on metabolism after reactivation of *Monascus*

Test indicators	*M. serorubescens* A1		P value	*M. lunisporas* A4		P value	*M. sanguineus* B1		P value
	Control	Experience		Control	Experience		Control	Experience	
Biomass (mg/mL)	21.86±0.98	22.45±1.32	0.567	9.30±0.75	10.11±1.18	0.374	37.28±1.13	36.79±1.30	0.646
Liquefying enzyme (10^3U/g)	2.36±0.30	2.34±0.13	0.915	6.99±0.61	6.67±0.71	0.587	1.13±0.05	1.14±0.04	0.973
Saccharifying enzyme (10^3U/g)	2.25±0.31	2.23±0.23	0.957	4.23±0.58	4.21±0.67	0.974	1.02±0.33	1.06±0.14	0.846
Pigment (U/mL)	44.08±2.69	42.63±3.04	0.567	38.00±1.31	39.39±3.13	0.545	109.50±2.29	108.50±2.05	0.603
γ - aminobutyric acid (μg/mL)	91.01±10.99	83.16±10.29	0.418	82.55±12.61	81.79±17.32	0.954	31.75±1.85	31.66±0.98	0.947
Lovastatin (μg/mL)	8.81±0.14	8.83±0.25	0.874	4.40±0.42	4.02±1.16	0.619	14.20±0.66	14.27±0.65	0.906
Citrinin (μg/mL)	5.29±0.68	4.92±0.55	0.506	0	0	-	8.87±0.79	8.15±0.25	0.208

Test indicators	*M. kaoliang* B6		P value	*M. argentinensis* B7		P value	*M. purpureus* C1		P value
	Control	Experience		Control	Experience		Control	Experience	
Biomass (mg/mL)	24.03±1.82	23.08±1.10	0.486	20.36±0.45	20.24±0.97	0.859	27.58±1.46	26.90±0.40	0.479
Liquefying enzyme (10^3U/g)	2.10±0.22	2.17±0.23	0.730	2.19±0.19	2.20±0.16	0.922	1.93±0.16	1.97±0.16	0.793
Saccharifying enzyme (10^3U/g)	1.65±0.15	1.69±0.06	0.722	1.31±0.17	1.30±0.13	0.937	1.57±0.17	1.59±0.22	0.917
Pigment (U/mL)	53.21±2.52	54.04±2.40	0.700	23.23±1.30	23.48±1.19	0.818	33.97±1.27	33.32±1.44	0.588
γ - aminobutyric acid (μg/mL)	109.48±15.17	107.93±12.32	0.897	104.45±3.73	104.17±3.99	0.933	111.56±4.75	113.78±2.90	0.528
Lovastatin (μg/mL)	6.63±0.72	6.63±0.31	0.991	2.32±0.33	2.33±0.67	0.990	2.95±0.51	2.89±0.52	0.900
Citrinin (μg/mL)	7.41±0.50	6.76±0.82	0.304	0	0	-	1.70±0.15	1.79±0.13	0.410

Notes: Metabolites were detected by shake flask fermentation with the modified potato dextrose water after reactivation of six *Monascus* species with PDA plate after different treatments. The experience group differed from the control group in whether they were treated with the selective medium containing 3.98% (v/v) lactic acid and 6.24% (v/v) ethanol.
